# Tolerance to Ceftriaxone in *Neisseria gonorrhoeae*: Rapid Induction in WHO P Reference Strain and Detection in Clinical Isolates

**DOI:** 10.3390/antibiotics11111480

**Published:** 2022-10-26

**Authors:** Margaux Balduck, Jolein Gyonne Elise Laumen, Saïd Abdellati, Irith De Baetselier, Tessa de Block, Sheeba Santhini Manoharan-Basil, Chris Kenyon

**Affiliations:** 1 HIV/STI Unit, Department of Clinical Sciences, Institute of Tropical Medicine, 2000 Antwerp, Belgium; 2 Laboratory of Medical Microbiology, University of Antwerp, 2610 Wilrijk, Belgium; 3 Clinical Reference Laboratory, Department of Clinical Sciences, Institute of Tropical Medicine, 2000 Antwerp, Belgium; 4 Division of Infectious Diseases and HIV Medicine, University of Cape Town, Anzio Road, Observatory 7700, South Africa

**Keywords:** *Neisseria gonorrhoeae*, antimicrobial resistance, tolerance, ceftriaxone, tolerance disc test

## Abstract

In addition to antimicrobial resistance, bacteria contain other mechanisms to survive antibiotic exposure such as tolerance, defined as the ability to slow metabolism by the extension of the lag phase without altering antimicrobial susceptibility. In a number of bacterial species, tolerance has been associated with treatment failure and infection chronicity and is found to precede and facilitate antimicrobial resistance. It is unknown if tolerance can be induced in *Neisseria gonorrhoeae*. In this study, we determined if tolerance to ceftriaxone (CRO) can be induced in *N. gonorrhoeae* and detected in clinical isolates. To induce tolerance, WHO P *N. gonorrhoeae* reference strain samples were grown under daily 3 h intermittent CRO exposure (10× the MIC), partitioned by overnight growth in GC broth. This cyclic exposure was performed for 7 consecutive days in sextuplicate, with two control cultures to which GC medium without antibiotics was added. To detect tolerance and assess CRO susceptibility, modified Tolerance Disc (TD) and Epsilometer tests were performed on isolates after each CRO exposure cycle. Additionally, this experiment was carried out on 18 clinical *N. gonorrhoeae* isolates. Tolerance was first detected after two CRO exposure cycles in five out of six samples. The phenotype differed per cycle with no clear pattern. No tolerance was found in control samples but was detected in 10 out of 18 clinical isolates. The present study is the first to demonstrate the induction of tolerance to CRO in *N. gonorrhoeae* through antibiotic exposure. In addition, tolerance to CRO was found in clinical samples.

## 1. Introduction

Gonorrhoea is the second most prevalent bacterial sexually transmitted infection (STI) worldwide and is caused by the obligate human pathogenic bacterium, *Neisseria gonorrhoeae* [1,2,3,4]. *N. gonorrhoeae* has acquired antimicrobial resistance (AMR) to multiple antibiotic classes, including decreased susceptibility to ceftriaxone (CRO), which is the current treatment advocated by most treatment guidelines [2,3,4,5]. An accurate understanding of the pathways between different types of antimicrobial exposure and AMR may help us to prevent the further emergence of resistance. For example, recent studies have found that antimicrobials used to treat other infections play an important role in the genesis of gonococcal AMR (bystander selection) [6,7]. 

Recent work in other bacterial species has found that antimicrobial-induced tolerance may be an additional pathway [8,9,10]. Tolerance is defined as the ability of bacteria to survive high bactericidal antibiotic exposure without an increase in minimum inhibitory concentration (MIC). They accomplish this primarily by slowing down their basic metabolism by extending the lag phase [10,11]. Under certain environmental challenges, tolerance may provide a survival advantage for bacteria. For example, under intermittent antibiotic exposure, the rapid development of high tolerance by extension of the lag phase, without the development of AMR, has been shown in a number of bacterial species to allow them to survive these pulses of antibiotic exposure [12,13,14,15]. 

In vitro antibiotic exposure experiments have shown that the induction of tolerance paves the way for the emergence of AMR [8,15,16,17,18,19]. In a number of these experiments, the development of AMR was always preceded by the emergence of tolerance [16,17,18,19]. One of the mechanisms for this effect is that tolerance provides a time window in which several resistance mutations can occur [8,12,15,20]. The effect of tolerance is of particular importance at high antibiotic concentrations, where it accelerates the fixation of partial resistance mutations [8,15,21].

Tolerance has also recently been shown to be clinically important. From a clinical standpoint, the few tolerant colonies that survive antibiotic therapy may be sufficient to multiply between doses of antimicrobials or once treatment has halted [12,14,22]. Tolerance could thus result in treatment failure [12,14,23]. Lazarovits et al. 2022 recently established that tolerance can be detected in *Escherichia coli* bloodstream isolates and is associated with an increased risk of reinfection, making tolerance an independent risk factor for repeated infection [24]. Tolerance has also been shown to play a role in the clinical persistence of *Pseudomonas aeruginosa* and *Mycobacterium tuberculosis* infections [16,17,18,19]. Although tolerance has been linked with treatment complications and failure, along with facilitated resistance development, it remains poorly characterized and is seldom considered in healthcare [12,14]. Moreover, to the best of our knowledge, no research on tolerance in *N. gonorrhoeae* has been conducted. 

A novel technique has been recently developed by Gefen et al. 2017 to detect the presence of tolerance, namely, the tolerance disc (TD) test. This is a modification of the standard Kirby–Bauer disc-diffusion assay [25]. During a disc-diffusion test, bacteria are plated with an antibiotic disc that creates an inhibition zone and allows the identification of non-susceptibility to the antibiotic [25,26]. However, this means that tolerant bacteria within the inhibition zone cannot be detected since, when the antibiotic concentration falls below the MIC, the nutrients in the agar plate will be depleted by the bacteria growing outside the inhibition zone. To bypass this nutrient depletion, during a TD test, the antibiotic disc is replaced by a nutrient disc after a set time, allowing regrowth of tolerant colonies [25]. Hence, a TD test can distinguish between susceptible, resistant, and tolerant bacteria [10,15,25]. 

This study investigated if: (1) tolerance to CRO could be induced in a WHO reference strain of *N. gonorrhoeae* via intermittent cyclic CRO exposure; (2) using a TD test, tolerance could be detected in clinical isolates of *N. Gonorrhoeae*; and (3) if the tolerance acquired by CRO induction could accelerate the acquisition of resistance to CRO.

## 2. Results

### 2.1. Ceftriaxone Tolerance Emerges Rapidly 

**Figure 1:**
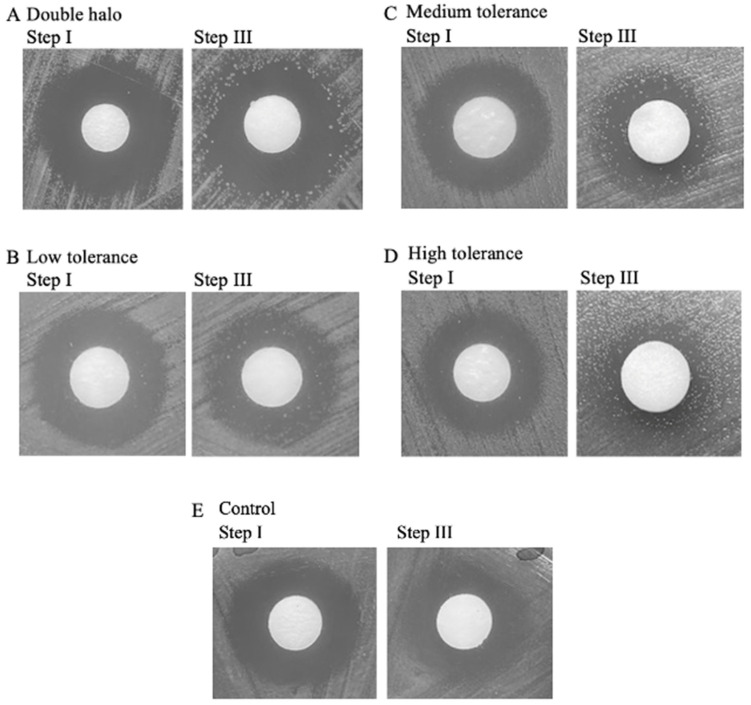
Induced tolerance in WHO P *N. gonorrhoeae* reference strain cyclically exposed to 3 h 1Ox CRO MIC, detected via a TD test. Step I: after overnight incubation with 0.008 µg CRO discs. Step III: results after nutrient replenishment, ranging from (**A**) a double halo appearance, (**B**) Low tolerance [0–10 colonies], (**C**) Medium tolerance [10–20 colonies], (**D**) High tolerance [>20 colonies], to (**E**) No tolerance; 6 mm discs.

A modified TD test protocol was performed on WHO P reference strain samples cyclically exposed to 10× the MIC for CRO for 3 h to detect the presence of induced tolerance (n = 6) [25]. Resuscitated tolerant colonies were detected in the inhibition zone, ranging in growth pattern. These were either observed as growth close to the edge of the inhibition zone, termed a double halo, or as dispersed colonies within the inhibition zone, labelled as low, medium, and high tolerance depending on the number of grown colonies (Table S1). The appearance of colonies was compared against the TD test results of control samples, where no growth was observed within the inhibition zone (Figure 1). 

**Figure 2:**
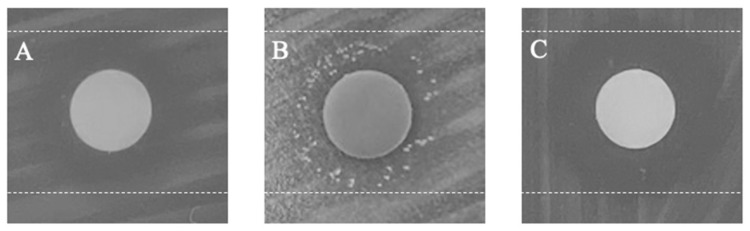
Follow-up TD test of tolerant colonies. (**A**) Zone of inhibition after step I of the TD test with 0.008 µg CRO. (**B**) Zone of inhibition after step III of the TD test. (**C**) Re-exposure of picked tolerant colonies to 0.008 µg CRO. Dashed lines mark the zone of inhibition, 6 mm discs.

Tolerant colonies that grew inside the inhibition zone and that were re-exposed to step I of the modified TD test generated an inhibition zone, comparable to that of the first TD test step (Figure 2). In addition, susceptibility testing confirmed no change in CRO MIC (Table S2). 

**Figure 3:**
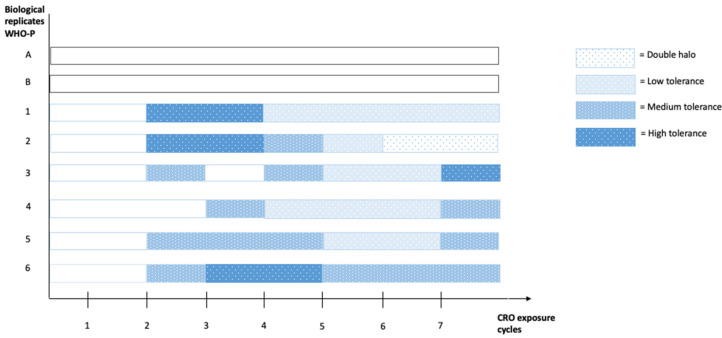
Evolution of the appearance of tolerance to CRO in WHO P reference *N. gonorrhoeae* strain (n = 6) after 3 h 10× MIC cyclic exposure to CRO, ascertained using a TD test. Samples A and B were control samples not exposed to CRO.

As depicted in Figure 3, tolerant colonies were observed in five out of six samples after two cycles of CRO exposure and after three cycles in the remaining sample. All samples simultaneously demonstrated tolerance after four cycles. Tolerance remained present in all samples from cycle 4 onwards. Noticeably, the tolerance level differed per cycle with no clear pattern evident. No tolerance was detected in the control samples (Samples A and B) throughout the experiment (Figure 3). 

### 2.2. Some Circulating Strains of N. gonorrhoeae Are Tolerant to Ceftriaxone 

**Figure 4:**
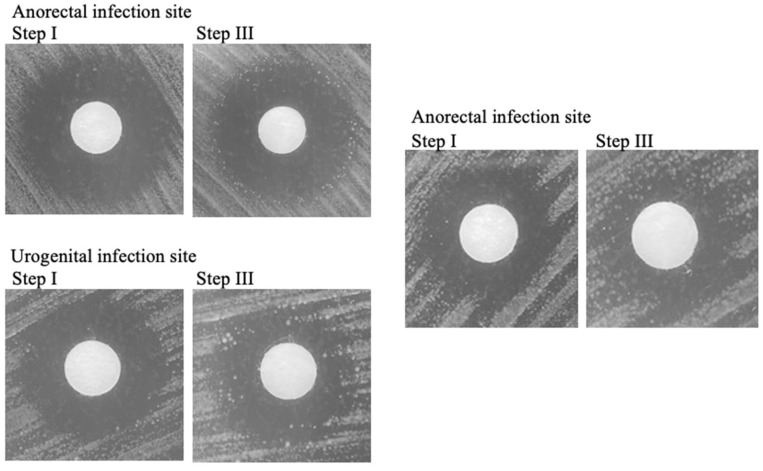
Detection of tolerance to CRO in three different clinical isolates of *N. gonorrhoeae* using a TD test. Step I: after overnight incubation with a 0.008 µg CRO discs. Step III: results after nutrient replenishment; 6 mm discs.

Tolerance was detected in 10 out of 18 clinical isolates (56%) using the above TD test protocol (Figure 4) [25]. The proportion of tolerance in isolates from the anorectum was seven out of nine (78%) and three out of nine (33%) in the urogenital isolates (Table 1) (Table S3). 

### 2.3. No Decreased Susceptibility to Ceftriaxone during Induction of Tolerance 

No increase in MIC for CRO was observed throughout the whole induction of tolerance experiment in the WHO P reference strain samples described in Section 2.2. More precisely, after each exposure cycle, the CRO MIC of each sample remained ≤ 0.008 µg/mL (Table S2). 

### 2.4. Tolerance Not Associated with Accelerated Induction of Ceftriaxone Resistance

All tolerant WHO P colonies were found to have a CRO MIC ≤ 0.008 µg/mL, comparable to the MIC of the control samples, determined using E-tests. No increase in CRO MIC was observed for either the tolerant colonies or the controls throughout the six subsequent crossover E-test experiments (Table S4). 

## 3. Discussion

This study established that tolerance to CRO can be induced in *N. gonorrhoeae* WHO P reference strain and can be found in clinical isolates of *N. gonorrhoeae*.

Interval high concentration CRO exposure of only two or three cycles was enough to induce the first appearance of a tolerant phenotype in WHO P reference strain of *N. gonorrhoeae*. In addition, tolerance remained present throughout the subsequent exposure cycles, without a change in CRO susceptibility (Figure 1 and Figure 3). In a similar vein, previous studies have shown that tolerance to ampicillin rapidly developed in *E. coli* samples exposed to intervals of high levels of ampicillin, without a change in MIC [8,14,27,28]. These cultures became tolerant to ampicillin by acquiring mutations encoding specifically for a tolerant phenotype, termed the tolerome, associated with an extended lag phase [8,14]. Similarly, Levin-Reisman et al. 2017 observed a growth appearance delay caused by an extended lag phase after three to four cycles of ampicillin exposure in multiple *E. coli* strains, without a rise in MIC, comparable to the time it took for tolerance to emerge in the current study [8]. 

We also detected tolerance in multiple clinical isolates of *N. gonorrhoeae* (Figure 4). Previous studies have detected tolerance in clinical isolates of other bacterial species, such as *E. coli* blood stream infections, respiratory isolates of *P. aeruginosa* in a cohort of cystic fibrosis patients, and various clinical isolates of *Staphylococcus aureus* [13,16,24]. These previous findings of tolerance in clinical samples were related to relapsing/repeat infections, signifying that, in addition to preceding and promoting resistance development, tolerance is an independent risk factor for relapsing/repeat infections [24]. It is not difficult to see how tolerance could emerge in a chronic infection exposed to multiple courses of antimicrobials over a period of years, such as with bronchiectatic *P. aeruginosa* infections in patients with cystic fibrosis [16,19]. The antimicrobial exposure that *N. gonorrhoeae* is subjected to in clinical practice is somewhat different as each *N. gonorrhoeae* infection is rapidly eliminated in most infections with the currently used highly efficacious treatments [29]. The finding of tolerance in clinical isolates may be related to three considerations. Firstly, *N. gonorrhoeae* is asymptomatic for most of the time that it circulates in a population. This means that antimicrobials, including cephalosporins and non-cephalosporin antimicrobials, used for any other indication may induce tolerance in this asymptomatic infection (bystander selection) [6]. Secondly, the prevalence of *N. gonorrhoeae* in Belgium is high in the higher-risk MSM population that contributed most of the clinical isolates we analyzed [2,30]. The prevalence of *N. gonorrhoeae* in this population typically exceeds 10%, where it mostly circulates asymptomatically [31]. This population is exposed to high volumes of antimicrobials [31,32]. As a result, when strains of *N. gonorrhoeae* spread within this sexual network, they become repeatedly subjected to high doses of CRO and other microbials, which could potentially lead to the development of tolerance [30,33]. Thirdly, the current study found that the proportion of *N. gonorrhoeae* isolates with tolerance was numerically higher in anorectal isolates compared to urogenital isolates (Table 1). No statistical analysis was performed on these results because the sample size was too small to make relevant conclusions. Anorectal infections differ from urethral isolates in a number of important respects. Whereas most urethral infections are symptomatic and have an infection duration of days to weeks, most anorectal infections are asymptomatic and persist for months (up to 6 months) [34]. There are several large differences between the anorectum and the male urethra. Whilst the urethra has an extremely limited microbiome, the anorectum, as part of the gastrointestinal tract (GIT), has the largest and most diverse microbial community in the human body [35]. The GIT mucosa differs from the urethral mucosal sites in other important respects. The complexity and abundance of the colonic microbiome means it requires a greater tolerance from the host immune system [36]. Tolerance here is defined as preventing the host immune system from evoking an excessive immune response to microbiota. A manifestation of this tolerance is that toll-like receptors on the apical surface of the GIT epithelium are strongly down-regulated [36]. There are also considerable interactions between the numerous species of bacteria in the GIT, which both down-regulate host inflammatory responses and influence the growth and phenotype of other bacterial species [37]. These features provide a plausible explanation for the longer duration of colonization and lack of symptoms found in anal compared to urethral *N. gonorrhoeae* [38]. Of note, several studies have found significant geno- and phenotypic differences between *N. gonorrhoeae* isolates from the urethra, cervix, and anorectum [38,39]. In particular, anorectal samples have been found to have a higher expression of the multiple transferable resistance (Mtr) efflux pump necessary to expel the hydrophobic molecules prevalent at this site [38]. Although speculative, these differences may also be sufficient to provide stronger selection pressure for the emergence of tolerance in *N. gonorrhoeae* in the anorectum compared to the urethra [38,39]. 

A key finding of this study was that tolerance could be detected through a modified TD test protocol, designed by Gefen et al. 2017, adjusted explicitly for *N. gonorrhoeae*. If the current findings can be confirmed with MDK99 analyses (minimum duration for killing 99% of the population), a TD test can be considered an adequate technique for tolerance detection in *N. gonorrhoeae*. Another use of the TD test could be to evaluate antibiotics that can effectively eliminate tolerant isolates of *N. gonorrhoeae* [25].

An additional finding was that tolerance did not accelerate the acquisition of CRO resistance. A previous study of *E. coli* found that tolerance preceded and facilitated the emergence of ampicillin resistance [8]. The discrepancy in AMR outcomes between this study and that of Levin-Reisman et al. 2017 might be due to the adopted experimental set-up of the present study. We used a protocol based on the work of Raisman et al. 2022. In their study, they were able to induce resistance to azithromycin in *N. elongata* by performing crossover E-tests [40]. In contrast, Levin-Reisman et al. 2017 induced AMR via continuing the cyclical intermittent antibiotic exposure set-up for up to 17 cycles [8,40]. An additional important consideration is that gonococcal resistance to CRO is more difficult to induce in vitro compared to AZM and ampicillin [41,42,43]. Gonococcal resistance to extended spectrum cephalosporins (ESCs) in circulating isolates typically occurs (amongst other mechanisms) via the acquisition of mutations in *penA*, typically in a stepwise fashion and frequently via horizontal gene transfer (HGT) from commensal *Neisseria* [41,42]. This type of HGT is not possible in the in vitro experimental set-up used in the current study. A previous study that attempted to induce CRO resistance in *N. gonorrhoeae* via a similar passaging strategy found that they could only induce resistance in one of six different strains used [44]. This strain acquired only one mutation in *penA* as well as several other mutations in other genes. These findings suggest that we should test the effect of gonococcal tolerance on the acquisition of resistance to other antimicrobials before we conclude that tolerance does not play a role in the genesis of AMR. It would be particularly interesting to test antibiotics such as CIP for which resistance mutations occur more easily and rapidly in vitro than CRO [45]. This could be achieved either with crossover E-tests or via cyclic antibiotic exposure experiments. 

Other limitations of this study included the small sample size of clinical isolates assessed and the fact that we only tested for tolerance to one antimicrobial. No confirmation of the TD test with another method for tolerance detection, such as MDK99 killing curves, was carried out, and no genotypic or transcriptome analyses were carried out. We were unable to explain the observed decreases in induced tolerance levels within single biological replicates. In addition, we did not compare gonococcal tolerance in vitro and in vivo. Further analyses, including whole genome sequencing and transcriptomics, are beyond the scope of the current study. Because the MICs were ascertained via E-tests, which can only assess MICs between 0.008 and 256 µg/mL, our methodology did not allow us to detect increases in MIC below 0.008 µg/mL. Despite these weaknesses, this was the first in vitro study to show that short exposure of *N. gonorrhoeae* to high concentrations of CRO can rapidly induce tolerance to CRO without a change in CRO susceptibility. Furthermore, the study established that tolerance could be ascertained via a relatively inexpensive and non-laborious TD test protocol adapted specifically to *N. gonorrhoeae*. Additionally, tolerance to CRO was detected in several clinical isolates of *N. gonorrhoeae*. 

## 4. Materials and Methods

### 4.1. Bacterial Strains

To test the ability of tolerance induction in *N. gonorrhoeae*, a WHO P reference strain with a CRO MIC of 0.004 µg/mL was used (highly susceptible to CRO) [46].

Clinical *N. gonorrhoeae* isolates (n = 18), highly susceptible to CRO (MIC ≤ 0.008 µg/mL), were analysed to detect the presence of tolerance in currently circulating strains of *N. gonorrhoeae*. These came from two sources. Firstly, we used all 14 isolates from the ResistAZM study (NCT05027516) that had a CRO MIC ≤ 0.008 µg/mL. This represented 56% of the isolates from this study (14/25). This single-centre study was an RCT that compared two therapies for gonorrhoea. Samples could be urethral, rectal, or pharyngeal. Secondly, we included the four most recent clinical isolates received by the Belgian National Reference Centre of STIs in 2022 with a CRO MIC ≤ 0.008 µg/mL. The MICs for CRO and ciprofloxacin (CIP) at baseline were determined via E-tests (BioMérieux, France). The site of infection was not considered during the sample selection (Table S3).

### 4.2. Induction of Tolerance to Ceftriaxone

To induce tolerance, six biological replicates of the WHO P reference strain (0.5–1.0 McF) were exposed to 10× the CRO MIC (0.04 µg/mL) for 3 h intervals in a cyclic manner, based on the protocol of Fridman et al. 2014 [14]. 

**Figure 5:**
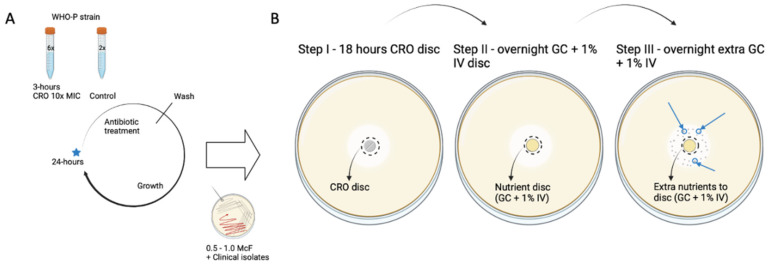
Induction of tolerance to CRO in WHO P *N. gonorrhoeae* reference strain and clinical isolates, detection via a CRO TD test. (**A**) Cyclic 3 h 10× MIC ceftriaxone interval exposure (n = 6) with two control samples not exposed to CRO. (**B**) TD test after every 24 h exposure cycle of reference strain samples (n = 6) including controls and clinical (n = 18) *N. gonorrhoeae* isolates with 0.008 µg CRO discs [14,25]. Blue star indicates the point at which cyclic exposure samples were inoculated, tested for MIC, and stored. Blue arrow indicates emergent tolerant colonies at Step III.

Every 24 h, overnight cultures were suspended in gonococcal broth medium (50 µL in 5 mL), containing double-distilled water supplemented with 15 g/L bacto protease peptone no. 3, 1 g/L soluble starch, 4 g/L K_2_HPO_4_ (174.18 g/mol), 1 g/L KH_2_PO_4_ (136.08 g/mol), 5 g/L NaCl (58.44 g/mol) and 1% BD BBL^TM^ IsoVitaleX, further referred to as GC medium. This medium was supplemented with a CRO solution to reach a concentration of 0.04 µg/mL (Merck Life Science, Darmstadt, Germany). All cultures were incubated for 3 h while placed on a roller-mixer on a maximum rotation of 80 rpm (RM 5, CAT, Staufen, Germany) at 36 °C in a 6.0% CO_2_ atmosphere. Following this step, antibiotics were twice washed off by 10 min centrifugation at 1400× *g* and cultures were resuspended (1:5) in fresh GC medium for overnight growth (21 h) on a roller-mixer at 36 °C and 6.0% CO_2_ [14]. After each exposure cycle, all samples were stored at −80 °C in skim milk containing 30% glycerol, see blue star Figure 1A. This cyclic CRO interval exposure experiment was performed for 7 consecutive days/cycles. Two control vials were included, with GC medium sans CRO (Figure 5A).

### 4.3. Tolerance Disc (TD) Test 

The CRO exposed WHO P reference strain and the clinical isolates were analysed using a modified TD test protocol (Gefen et al. 2017) to detect the presence of tolerance to CRO [25]. 

After each 24 h CRO exposure cycle (blue star Figure 5A), all WHO P cultures (n = 6) and control samples, were inoculated on BDTM Columbian Blood Agar with 5% Sheep Blood for overnight growth and then subjected to a TD test, adjusted to *N. gonorrhoeae* and CRO specifically by placing a 0.008 µg CRO antibiotic disc on each inoculated BD^TM^ GC agar plate (step I) [25]. The concentration of CRO in the disc was calculated in such a way that the concentration of CRO within the inhibition zone would fall below the MIC after overnight incubation. The discs were created by soaking 6 mm blank discs (Merck Life Science, Darmstadt, Germany) in 25 µL of the required amount of CRO (Merck Life Science, Darmstadt, Germany). After approximately 18 h of incubation at 36 °C and 6.0% CO_2_, CRO discs were replaced by nutrient discs created by soaking 6 mm blank disks in a 25 µL GC medium and left for an additional night of incubation (step II). Contents of nutrient discs differed from those described in the original TD test protocol to provide nutrients that support *N. gonorrhoeae* growth [25]. Specifically, an additional 10 µL of GC medium was added to the same nutrient disc and plates were incubated for one more night (step III) since *N. gonorrhoeae* tolerant colonies required an additional night of incubation before their emergence [25]. If tolerant colonies were observed after 48 h with a nutrient disc (blue arrow Figure 5B), they were selected, inoculated on blood agar plates, and stored in 30% glycerol skim milk at −80 °C. Based on the location and number of tolerant colonies that appeared, TD test results were categorized into tolerance sub-categories: double halo (growth around inhibition zone), low tolerance (0–10 colonies), medium tolerance (10–20 colonies), and high tolerance (> 20 colonies) [25]. All TD test results were examined by two independent assessors and photographed. The above-mentioned protocol was also used to detect tolerance in the clinical isolates (n = 18) after being revived from −80 °C storage in skim milk containing 30% glycerol (Figure 5B). 

### 4.4. Susceptibility Testing 

Following each CRO exposure cycle, the MIC of all reference strain samples was determined using a CRO gradient MIC strip/E-test ranging from 0.016 µg/mL to 256 µg/mL (BioMérieux, France). All were carried out in compliance with the manufacturer’s instructions [47]. 

### 4.5. Induction of Ceftriaxone Resistance 

To analyse whether tolerance accelerates the development of CRO resistance, selective pressure was applied on tolerant colonies obtained through cyclic CRO exposure of WHO P reference strain (n = 13) and non-tolerant sensitive strains (n = 4), in parallel, using a modified cross-plating protocol of Raisman et al. 2022 [40]. 

**Figure 6:**
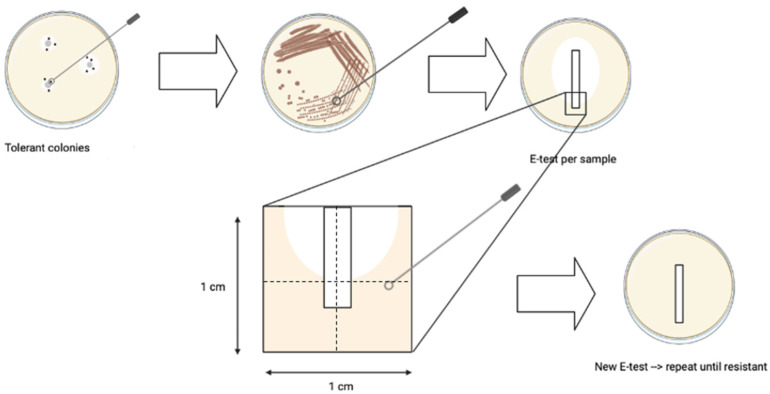
Parallel in vitro evolution of *N. gonorrhoeae* CRO tolerant colonies (n = 13) and non-tolerant sensitive cultures (n = 4) under selective pressure to induce CRO resistance through 7 consecutive crossover CRO E-tests.

Selected tolerant colonies included those obtained after the first cycle, where tolerance appeared for each sample individually and after the last (7th) CRO exposure cycle in the experiments detailed in Section 4.2. Non-tolerant sensitive controls consisted of the 2 control cultures from the cyclic CRO exposure taken after the 7th/last exposure cycle, in duplicate. Bacterial suspensions (0.5–1.0 McF) were made from overnight cultures of selected samples in PBS, revived from −80 °C stored colonies, and used to inoculate a BD^TM^ GC agar plate. Next, a ceftriaxone gradient MIC strip/E-test ranging between 0.016 µg/mL and 256 µg/mL (BioMérieux, France) was placed on all the plates. After overnight incubation at 36 °C and 6.0% CO_2_, a standardized amount of culture was taken from each plate, using a 1 cm^2^ stencil placed at a certain position of the E-test strip as indicated in Figure 6. Subsequently, this culture was suspended in PBS and re-inoculated on a new GC agar plate with a new E-test. This method was repeated every 24 h for all samples for 7 consecutive days (Figure 6). 

## Figures and Tables

**Table 1:** Tolerance appearance (yes or no) in anorectal and urogenital clinical *N. gonorrhoeae* isolates, with corresponding proportions.

	Tolerance		
	YES	NO	Proportion
Anorectal	7	2	78%
Urogenital	3	6	33%

## Supplementary Materials

The following supporting information can be downloaded at: https://www.mdpi.com/article/10.3390/antibiotics11111480/s1, Table S1: Tolerant colonies count of *N. gonorrhoeae* strains (n = 6) and two controls (A and B) cyclically exposed to ceftriaxone (CRO). Table S2: No change in ceftriaxone minimum inhibitory concentration of *N. gonorrhoeae* strains (n = 6) and two controls (A and B) cyclically exposed to ceftriaxone, determined through susceptibility testing via E-tests.; Table S3: The minimum inhibitory concentration and susceptibility/resistance of used WHO P reference and clinical *N. gonorrhoeae* strains for ceftriaxone, ciprofloxacin, and azithromycin. Plus, site of infection and tolerance detection of clinical isolates and gender of patients; Table S4: Tolerance not associated with accelerated induction of ceftriaxone resistance. Ceftriaxone minimum inhibitory concentration *of N. gonorrhoeae* tolerant colonies (n = 13) compared to controls (n = 4) under in vitro selective pressure via 7 consecutive crossover ceftriaxone E-test.
