# Supplementary material for: Tolerance to Ceftriaxone in Neisseria gonorrhoeae: Rapid Induction in WHO P Reference Strain and Detection in Clinical Isolates
Source: Antibiotics (Basel). 2022 Oct 26;11(11):1480. doi: 10.3390/antibiotics11111480 (PMC9686967; doi:10.3390/antibiotics11111480)
Supplement: Supplementary file 1 [file antibiotics-11-01480-s001.zip › Supplementary materials.pdf]

Supplementary materials "Tolerance to ceftriaxone in *Neisseria gonorrhoeae*: rapid induction in WHO P reference strain and detection in clinical isolates".

Table S1: Tolerant colonies count of *N. gonorrhoeae* strains (n = 6) and two controls (A and B) cyclically exposed to ceftriaxone (CRO).

	Tolerant colonies count						
**Samples**	**1**	**2**	**3**	**4**	**5**	**6**	**7**
**A**	/	/	/	/	/	/	/
**B**	/	/	/	/	/	/	/
**1**	/	83	67	8	6	7	9
**2**	/	72	78	18	8	/	/
**3**	/	18	/	16	7	8	66
**4**	/	/	19	7	5	8	18
**5**	/	15	18	16	7	6	20
**6**	/	14	92	83	17	13	19

Table S2: No change in ceftriaxone minimum inhibitory concentration (MIC) of *N. gonorrhoeae* strains (n = 6) and two controls (A and B) cyclically exposed to ceftriaxone (CRO), determined through susceptibility testing via E-tests.

	CRO MIC (µg/mL)/CRO exposure cycle						
**Samples**	**1**	**2**	**3**	**4**	**5**	**6**	**7**
**A**	<0.008 (S)	<0.008 (S)	<0.008 (S)	<0.008 (S)	<0.008 (S)	<0.008 (S)	<0.008 (S)
**B**	<0.008 (S)	<0.008 (S)	<0.008 (S)	<0.008 (S)	<0.008 (S)	<0.008 (S)	<0.008 (S)
**1**	<0.008 (S)	<0.008 (S)	<0.008 (S)	<0.008 (S)	<0.008 (S)	<0.008 (S)	<0.008 (S)
**2**	<0.008 (S)	<0.008 (S)	<0.008 (S)	<0.008 (S)	<0.008 (S)	<0.008 (S)	<0.008 (S)
**3**	<0.008 (S)	<0.008 (S)	<0.008 (S)	<0.008 (S)	<0.008 (S)	<0.008 (S)	<0.008 (S)
**4**	<0.008 (S)	<0.008 (S)	<0.008 (S)	<0.008 (S)	<0.008 (S)	<0.008 (S)	<0.008 (S)
**5**	<0.008 (S)	<0.008 (S)	<0.008 (S)	<0.008 (S)	<0.008 (S)	<0.008 (S)	<0.008 (S)
**6**	<0.008 (S)	<0.008 (S)	<0.008 (S)	<0.008 (S)	<0.008 (S)	<0.008 (S)	<0.008 (S)

Table S3: The minimum inhibitory concentration (MIC) and susceptibility/resistance (S/R) of used WHO P reference and clinical *N. gonorrhoeae* strains for ceftriaxone (CRO), ciprofloxacin (CIP), and azithromycin (AZI) [46]. Plus, site of infection (anorectal – A, urogenital – U) and tolerance detection (yes – Y or no – N) of clinical isolates and gender of patients (male – M, female - F).

	MIC (µg/mL)			Infection site	Tolerance	Gender
**Strain**	**CRO**	**CIP**	**AZI**			
**WHO P**	0.004 (S)	0.004 (S)	4 (R)	/	/	
**RA00002**	< 0.008 (S)	0.5 (R)	0.094 (S)	A	Y	M
**RA00003**	< 0.008 (S)	0.032 (S)	2 (R)	A	Y	M
**RA00007**	< 0.008 (S)	0.023 (S)	2 (R)	A	Y	M
**RA00012**	< 0.008 (S)	0.003 (S)	0.094 (S)	U	N	M
**RA00015**	< 0.008 (S)	1.5 (R)	0.19 (S)	A	Y	M
**RA00018**	< 0.008 (S)	3 (R)	0.094 (S)	U	Y	M
**RA00023**	< 0.008 (S)	0.023 (S)	2 (R)	U	Y	M
**RA00024**	< 0.008 (S)	1.5 (R)	0.19 (S)	U	N	M
**RA00029**	< 0.008 (S)	0.002 (S)	0.19 (S)	A	Y	M
**RA00032**	< 0.008 (S)	0.023 (S)	1.5 (R)	A	N	M
**RA00035**	< 0.008 (S)	0.006 (S)	2 (R)	A	Y	M
**RA00045**	< 0.008 (S)	0.006 (S)	1.5 (R)	A	Y	M
**RA00069**	< 0.008 (S)	0.75 (R)	0.19 (S)	U	N	M
**RA00071**	< 0.008 (S)	2 (R)	1 (S)	A	N	M
**22227**	< 0.008 (S)	0.38 (S)	0.002 (S)	U	N	M
**22235**	< 0.008 (S)	0.094 (S)	0.002 (S)	U	N	M
**22249**	< 0.008 (S)	0.125 (S)	0.003 (S)	U	N	M
**22253**	< 0.008 (S)	0.125 (S)	0.002 (S)	U	Y	F

Table S4: Tolerance not associated with accelerated induction of ceftriaxone resistance. Ceftriaxone minimum inhibitory concentration of *N. gonorrhoeae* tolerant colonies (n = 13) compared to controls (n = 4) under *in vitro* selective pressure via 7 consecutive crossover CRO E-test.

	**CRO MIC colonies/crossover E-test (µg/mL)**						
	**1**	**2**	**3**	**4**	**5**	**6**	**7**
**Tolerant colonies**							
1	<0.008 (S)	<0.008 (S)	<0.008 (S)	<0.008 (S)	<0.008 (S)	<0.008 (S)	<0.008 (S)
2	<0.008 (S)	<0.008 (S)	<0.008 (S)	<0.008 (S)	<0.008 (S)	<0.008 (S)	<0.008 (S)
3	<0.008 (S)	<0.008 (S)	<0.008 (S)	<0.008 (S)	<0.008 (S)	<0.008 (S)	<0.008 (S)
4	<0.008 (S)	<0.008 (S)	<0.008 (S)	<0.008 (S)	<0.008 (S)	<0.008 (S)	<0.008 (S)
5	<0.008 (S)	<0.008 (S)	<0.008 (S)	<0.008 (S)	<0.008 (S)	<0.008 (S)	<0.008 (S)
6	<0.008 (S)	<0.008 (S)	<0.008 (S)	<0.008 (S)	<0.008 (S)	<0.008 (S)	<0.008 (S)
7	<0.008 (S)	<0.008 (S)	<0.008 (S)	<0.008 (S)	<0.008 (S)	<0.008 (S)	<0.008 (S)
8	<0.008 (S)	<0.008 (S)	<0.008 (S)	<0.008 (S)	<0.008 (S)	<0.008 (S)	<0.008 (S)
9	<0.008 (S)	<0.008 (S)	<0.008 (S)	<0.008 (S)	<0.008 (S)	<0.008 (S)	<0.008 (S)
10	<0.008 (S)	<0.008 (S)	<0.008 (S)	<0.008 (S)	<0.008 (S)	<0.008 (S)	<0.008 (S)
11	<0.008 (S)	<0.008 (S)	<0.008 (S)	<0.008 (S)	<0.008 (S)	<0.008 (S)	<0.008 (S)
12	<0.008 (S)	<0.008 (S)	<0.008 (S)	<0.008 (S)	<0.008 (S)	<0.008 (S)	<0.008 (S)
13	<0.008 (S)	<0.008 (S)	<0.008 (S)	<0.008 (S)	<0.008 (S)	<0.008 (S)	<0.008 (S)
14	<0.008 (S)	<0.008 (S)	<0.008 (S)	<0.008 (S)	<0.008 (S)	<0.008 (S)	<0.008 (S)
**Controls**							
1	<0.008 (S)	<0.008 (S)	<0.008 (S)	<0.008 (S)	<0.008 (S)	<0.008 (S)	<0.008 (S)
2	<0.008 (S)	<0.008 (S)	<0.008 (S)	<0.008 (S)	<0.008 (S)	<0.008 (S)	<0.008 (S)
3	<0.008 (S)	<0.008 (S)	<0.008 (S)	<0.008 (S)	<0.008 (S)	<0.008 (S)	<0.008 (S)
4	<0.008 (S)	<0.008 (S)	<0.008 (S)	<0.008 (S)	<0.008 (S)	<0.008 (S)	<0.008 (S)
